# Astrocytic Insulin-Like Growth Factor-1 Protects Neurons Against Excitotoxicity

**DOI:** 10.3389/fncel.2019.00298

**Published:** 2019-07-09

**Authors:** Wei Chen, Bin He, Wusong Tong, Jinsong Zeng, Ping Zheng

**Affiliations:** ^1^Department of Neurosurgery, Shanghai University of Medicine and Health Sciences, Shanghai, China; ^2^Department of Neurosurgery, Shanghai Pudong New Area People’s Hospital, Pudong, China

**Keywords:** insulin-like growth factor-1, kainic acid, excitotoxicity, hyperphosphorylated tau, astrocytes, neurons

## Abstract

**Background:**

Exogenous insulin like growth factor-1 (IGF-1) is known to be neuroprotective in animal models with brain insults, while it can also cause hyperexcitability in rodents. In this regard, the role of endogenous IGF-1 in brain responses to brain insults like excitotoxicity, a common pathology in brain injuries, remains to be elucidated. Here, we investigated the potential role of cell-specific endogenous IGF-1 in the kainic acid (KA) -induced degeneration of the neurons.

**Methods:**

Kainic acid was given to primary cultured cortical neurons and co-cultured astrocytes were added as a supportive system. We evaluated the cell proliferation rate, IGF-1 level in different groups and applied the PCR-Chip assay to explore the downstream of IGF-1. In addition, we applied the viral transfer of astrocytic IGF-1 to rodents treated with KA and assessed the associated molecular marker and behavioral outcomes in these rodents.

**Results:**

We found KA induced increased cell death and hyperphosphorylated tau in neurons; co-cultured astrocytes could prevent these pathologies, and this rescuing effect was abrogated with blockade of the astrocytic IGF-1 with AG1024 (IGF-1R inhibitor). PCR-Chip assay identified that astrocytic IGF-1 could decrease the p-GSK-3 at Tyr 216 in neurons treated with KA and this effect was abrogated with AG1024 as well. In addition, *in vivo* study showed that gene transfer of astrocytic IGF-1 decreased p-tau and cognitive dysfunction in KA mice.

**Conclusion:**

Our results show astrocytic IGF-1 exhibits neuroprotective properties in neurodegenerative processes in the CNS.

## Introduction

Excitotoxicity is involved in neurodegenerative diseases such as traumatic brain injury, stroke and epilepsy. Following neurotoxic lesions such as ischemia (Aberg et al., 2006), or injury to the cortex (Pansiot et al., 2016) or the spinal cord (Sisti et al., 2019), insulin growth factor-1 (IGF-1), and its receptors are found to increase in associated brain lesions, implying a role of IGF-1 in the pathological response to these brain insults. IGF-1 has been shown to exert its neuroprotective effects on different cells after brain injury.

Insulin like growth factor-1 is demonstrated to be neuroprotective after brain trauma or stroke. In a traumatic brain injury (TBI) rat model, IGF-1 is shown to increase in the ipsilateral side of the brain injury (Santi et al., 2018). In addition, exogenous IGF-1 has been found to protect the cerebral parenchyma in stroke models and to result in improved neurological outcomes (Liang et al., 2018). Generally, IGF-1 combines with IGF-1 receptor (IGF-1R) to exert functions. IGF-1R is widely distributed in both neurons and astrocytes in the brain. Neuronal IGF-1R is thought to affect neuronal polarity during cerebral cortical migration (Gontier et al., 2015), while reduced IGF1 signaling in astrocytes impairs their support for neurons under conditions of stress and this is associated with defects in the mitochondrial respiratory chain in astrocytes (Nieto Guil et al., 2017). In translational sessions, researchers found exogenous IGF-1 decreases brain lesions and prevents neurological deficits in a rodent stroke model (Liu et al., 2004).

Moreover, the pharmaceutical role of IGF-1 has been confirmed in other animal models of neurodegeneration, such as multiple sclerosis (Akcali et al., 2017), Alzheimer’s (Zheng and Tong, 2017) and Parkinson’s disease (Xiao et al., 2017), which have a common neuropathology in excitotoxicity. Most interestingly, IGF-1 has been tested both in preclinical (Yao et al., 2008) and in open-label clinical trials for the treatment of cerebellar ataxia with encouraging results (Sanz-Gallego et al., 2014). By contrast, several controversial studies have reported exogenous IGF-1 can induce cellular hyperexcitability regardless of neuronal protection *(12)*. A potential reason for the obviously contradictory findings can be that different responses of IGF-1 to excitotoxicity are cell-dependent (Quesada et al., 2007). Until now, few studies have been specific in this aspect. In addition, astrocytes are shown to protect neurons in response to excitotoxicity (Chen et al., 2019). In this way, it would be interesting to investigate the effect of cell-specific endogenous IGF-1, and we report here that co-cultured astrocytes protect neurons against excitotoxicity and, very importantly, gene delivery of IGF-1 in astrocytes shows a neuroprotection *in vivo* following excitotoxicity.

## Materials and Methods

### Animals and Reagents

We used postnatal Sprague-Dawley rats for *in vitro* cultures (P3 days for astrocytes and E18 for neurons). 40 C57^*^B6 mice at 6–8 weeks old were used for *in vivo* study (10 for each group). All experiments were carried out based on medical ethics guidelines (20170223-001). KA and AG1024 (IGF-1R antagonist) were purchased from Sigma (Steinheim, Germany).

### Cell Culture

Cortical cultures were obtained from either E18 or P3 rats (P3 days for astrocytes and E18 for neurons). In brief, harvested cerebral cortex was digested in HBSS and re-suspended in Neurobasal medium supplemented with 2% B-27 and 1% Glutamine to inhibit the glial division. Cells were plated onto 6 dishes coated with poly-l-lysine (1 μg/ml) at a final density of 1.5 × 10^6^/well for neurons or 0.45 × 10^6^/well for astrocytes according to previous reports *(14)*. The cultured neurons showed neurite extensions after 5–7 days (Supplementary Figure S1). On the day of the experiment, KA at doses of 0.1, 1, and 10 μmol/L was added to the medium. We applied KA as an excitoxic stimulus due to our previous reports that it can hyperphosphorylated tau and result in neurodegeneration *(15)*. Astrocyte were taken from P3 rats. Cells were grown in DMEM-F12 medium. Co-cultures of astrocyte and neurons were performed in a transwell system, as previously described in detailed reports. We treated neurons with KA at doses of 0.1, 1, and 10 μmol/L for 8 h in the down chamber, while cocultured astrocytes stayed in the upper chamber. Three repeated experiments were carried out in duplicate wells.

### Thiazolyl Blue Tetrazolium Bromide Assay (MTT) – Cell Viability

We applied the MTT assay to assess the cell viability according to a previous report *(5)*. Viability of vehicle-treated control cells without KA exposure was taken as standard, with optical density value determined on the fluorescence reader.

### Brain Excitotoxicity

Four weeks after the virus injection, 6- to 8-week-old male mice (10 per group) were given one ip injection with KA (5 mg/KG) and the same amount of PBS was administered to controls. Following surgery, mice were returned to their cages, kept at room temperature and allowed free access to food and water. After 6 weeks from the injection, we analyzed the p-tau levels in hippocampus and cortex in experimental mice.

### Sample Preparation and Western Blot Analysis

Western blotting was performed as described. Cells were washed once with ice-cold PBS and artificial cerebro-spinal fluid (Sigma-Aldrich, CA, United States). To normalize for protein load, membranes were reblotted (Re-Blot, Chemicon, United States) and incubated with an appropriate control antibody (see section “Results”). BSA method was applied to quantify the basic expression of loading proteins (Bio-Rad, United States). Blotting images were carried out using Image J (MIT, Boston, MA, United States). The representative image is taken from three repeated trials. Most western blot studies were in neurons, except IGF-1 analysis in astrocytes.

### Immunofluorescence

Animals were perfused transcardially with dPBS (Thermo Fisher Scientific, MA, United States) followed by 4% paraformaldehyde. The brains were removed from the cranial vault and post fixed in 4% paraformaldehyde overnight at 4°C. Brains were transferred to 15% sucrose overnight at 4°C and subsequently embedded in Tissue-teq (OCT, MA, United States) and then sectioned (20 microns) using a cryostat (Microm HM550, Thermo Fisher Scientific, MA, United States).

Sections were collected on superfrost slides (Thermo Fisher Scientific, MA, United States), air dried, and fixed with 4% paraformaldehyde for 30 min and blocked in blocking buffer (2% normal goat serum or rabbit serum and 0.2% triton X-100 in dPBS) for 1 h at room temperature. Sections were then incubated with primary antibodies for hIGF-1 (Sigma, 1:80 dilution) or GFAP (Sigma, CA, United States, 1:80 dilution) overnight for 16 h, followed by a 1 h incubation with fluorescent-labeled secondary antibodies (Oregon green 488 donkey anti-goat, 1:500 dilution and Oregon green 488 goat anti-rabbit, 1:500 dilution, respectively). Fluorescent labeling was visualized on the Nikon ZR1500 microscopy and captured digitally by Nikon customized software (Waltham, MA, United States).

As for quantitative assessment of neuronal loss (NeuN) and PS-198, images (10 times) were captured from coronal sections at the ipsilateral cortex by a researcher who was blinded to the experimental conditions. With Image J software (NIH, United States), 16-bit pictures were transferred manually to quantify the mean optical density of fluorescence of each sample. For GFAP and IGF-1 fluorescence, we did a colocalization study to check the expression of IGF-1 in astrocytes as we used a GFAP-promoter for *in vivo* virus transduction as previously reported (Okoreeh et al., 2017).

### Antibodies

Goat anti-IGF-1 antibody (ab106836), rabbit anti-ps198 antibody (ab79540), chicken anti-NeuN antibody (ab134014), and rabbit anti-GFAP (ab33922) were purchased from Abcam (Cambridge, MA, United States). Rabbit Polyclonal GSK3 Beta Antibody (22104-1-AP) was purchased from Proteintech (Cambridge, CA, United Kingdom). Rabbit Anti-phospho-GSK3 Beta (Tyr216) antibody (bs-4079R) was provided by Bioss (Danvers, MA, United States). Donkey anti goat HRP and goat anti rabbit HRP antibody (PAB0012 and PAB0011) were purchased from Bioswamp (Waltham, MA, United States). For secondary antibodies used in immunofluorescence, we used the Alexa Fluor 488 and 594 from Abcam (Cambridge, MA, United States).

### PI3K-Akt PCR Array

An RT2 Profiler PI3K-Akt PCR Array (WCgene, Biotechnology, Shanghai) was utilized to screen a battery of downstream factors of IGF-1 in neurons according to previous reports (Chen et al., 2019).

### Gene Transfer and Virus Constructs

Recombinant adeno associated virus serotype 8 (AAV2/8) was packaged (Obio, Shanghai) with the open reading frame (ORF) of human (h) IGF-1 gene downstream of the astrocyte-specific promotor, GFAP (GFAP-AAV8-hIGF-1). This construct contained the EGFP reporter gene as well under the GFAP promoter to visually detect those transfected cells. The control construct is composed of an identical shuttle vector without the hIGF-1 gene (GFAP-AAV8-control).

Animals were anesthetized (medical oxygen 3% and isoflurane 1.5%) and fixed in a stereotaxic device (RWD Instruments, Shanghai). One small hole was drilled into the skull for the Bregma reference of lateral ventricle: 2 mm posterior, 1.5 mm lateral, and a depth of 2.5 mm beneath the dura. In each case, a Hamilton syringe with an injection needle touched the associated region and the virus was gradually injected into the lateral ventricle at the rate of 0.25 μl/min for a total of 2 ml at around 8–10 min. All animals received one injection with a left side of the brain with either the GFAP-AAV8-hIGF-1 or the GFAP-AAV8-control construct. Animals were cared for 4 weeks to allow recovery and permit the viral expression in associated brain areas, followed by the KA or saline injection.

### Behavior Tests

Modified limb preference (MLPT) test is applied to test the motor function of rodents with a scale from 0 to 5 in three items. The higher score means more severe motor function, and normal mice show zero. First, the rat is suspended 10 cm above a table, and the stretch of the forelimbs toward the table is observed and evaluated: a normal stretch is scored as 0 points; abnormal flexion is scored as 1 point. Next, the rat is positioned along the edge of the table, with its forelimbs suspended over the edge, and is then allowed to move freely. Each forelimb (forelimb-second task, hindlimb-third task) is gently pulled down, and retrieval and placement are evaluated. Finally, the rat is placed near the table edge, in order to assess the lateral placement of the forelimb. The three tasks are scored in the following manner: normal performance is scored as 0 points; delayed (at least 2 s) and/or incomplete performance is scored as 1 point; no performance is scored as 2 points. Total score 5 points indicate maximal neurological deficit, and a score of 0 points denotes normal performance (Lee et al., 2008).

Y-Maze is used to assess the cognitive function of rodents. As previously reported, Y-maze experiments were performed in a Y-shaped device with three arms of the same size (38 cm in length, 8 cm in width, and 13 cm in height; San Diego Instruments, San Diego, CA, United States). A visual cue with different figures was put above the distal end of each arm. First, mice underwent a training session for 15 min. During this session, a novel arm was initially blocked, and then the mouse was placed in the distal end of one arm and allowed to freely explore the two arms for 15 min. After a 2-h recovery interval, all mice underwent a testing session for 5 min. In the testing session, the novel arm was open, and the mouse was put in the same end of the previous arm and permitted to freely explore all three arms for 5 min. The arm and visual cues were randomized between, but not within mice. An overhead camera recorded each trial, and the time spent into each one of the arms was quantified using Ethovision tracking software (Noldus, Wageningen, Netherlands). Shorter duration in novel arms demonstrates cognitive dysfunction for rodents.

### Statistical Analysis

Experimental data were mostly expressed as mean ± SEM, and statistical differences were compared by two-way ANOVA followed or a one-way ANOVA in different studies with GraphPad Prism software (La Jolla, CA, United States). Differences with a *p*-value <0.05 were considered statistically different.

## Results

### KA Induced Neuronal Death in a Concentration-Dependent Manner

**FIGURE 1:**
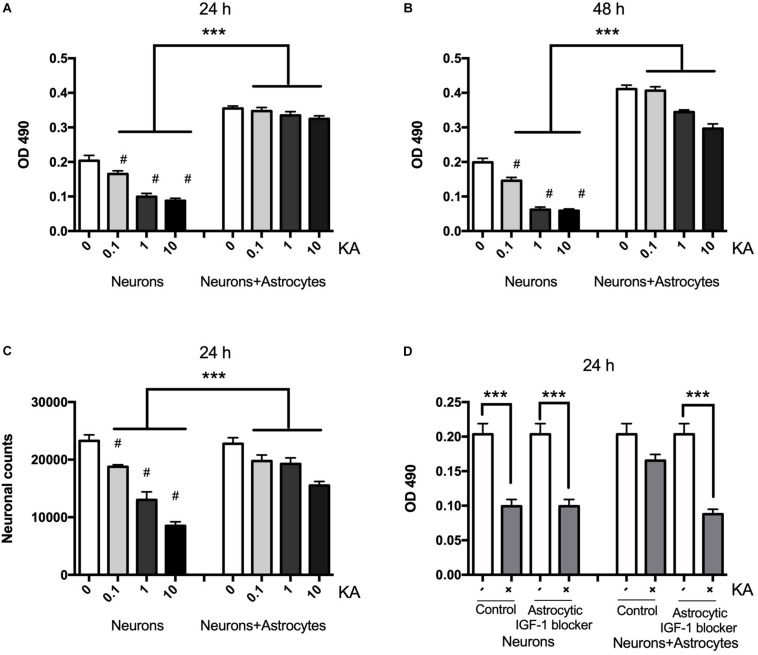
Neurons are protected from excitotoxic injury in the presence of astrocytes whereas when cultured alone they rapidly die with a dose-response effect. Viability of neurons was measured by MTT at OD 490 1 and 2 days after KA treatment in the presence or absence of wild type astrocytes (**A,B**, *F*_2__,__24_ = 78.35, ^∗∗∗^*P* < 0.0001 and *F*_2__,__24_ = 294.2, ^∗∗∗^*P* < 0.0001, neurons+KA vs. neurons+astrocytes+KA; ^#^*P* < 0.0001 compared to neurons in KA_0_ group, *n* = 6 in each group). Cell counts from KA treated or saline showed that KA increased neuronal loss at 24 h with a dose-dependent manner (**C**, ^∗∗∗^*P* < 0.05, neurons+KA vs. neurons+astrocytes+KA; ^#^*P* < 0.05 compared between neurons+KA and cocultured neurons+KA at the same KA concentration, *n* = 3 in each group). IGF-1 blocker does not change KA inducing neuronal loss, but it diminishes the neuroprotective effect of astrocytic IGF-1 in the coculture system (**D**, *F* = 8.90; ^∗∗∗^*P* < 0.05 compared with non-KA group).

Neuronal death assessed with MTT is shown to be gradually increased after KA treatment for 8 h ranging from 0.1 to 10 μM when compared with the controls at concentrations post-treatment using MTT and cell counting (Figures 1A,B). The neuronal viability was reduced from the dose of 0.1 μM (*P* < 0.05, *n* = 6), and when the dose was increased to over 10 μM, the viability decreased significantly to 45 ± 5.89% (*P* < 0.01, *n* = 6). KA treatment also decreased the neuronal counts between coculture group and neuron alone group from 19750 to 18750, 19250 to 13000, 15500 to 8500/mm^3^, when KA treatment at 0.1, 1, and 10 μmol/L, respectively (*P* < 0.05, Figure 1C). In the coculture system with different KA treatment groups, there is no difference in OD value at 24 h (*P* > 0.05); however, KA treatment at 1 and 10 μmol/L could reduce the OD value in neurons even in the co-culture system at 48 h after the KA treatment. This might indicate that the supportive function of astrocytes is transient. To confirm the different role of neuronal and astrocytic IGF-1, we specifically blocked the astrocytic IGF-1 with AG1024 (IGF-1R inhibitor differently added in the neuronal or astrocyte medium) in co-culture medium. We found there was no effect of astrocytic IGF-1 blocker in sole cultured neurons with KA (1 μmol/L for 8 h), but it diminished the neuroprotective effect of astrocytic IGF-1 in the co-culture system. Hence, endogenous production of IGF-1 from astrocyte but not neurons is necessary and sufficient to protect neurons (Figure 1D).

### Astrocytic IGF-1 Signaling Is Essential in Astrocyte Neuroprotection Against Neuronal Excitotoxicity

**FIGURE 2:**
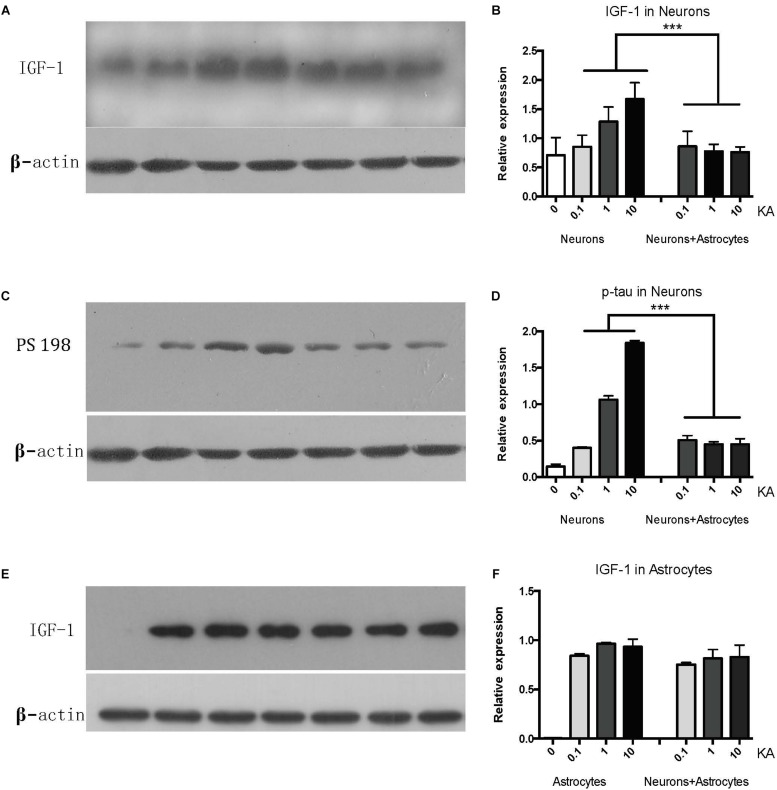
The neuronal IGF-1 and phosphorylated tau (PS198) increase in response to KA with a dose-response effect while it remains lower level in the presence of astrocytes **(A–D)**. The astrocytic IGF-1 was secreted substantial and a dose-independent manner after KA **(E,F)**. ^∗∗∗^*P* < 0.05, compared between neurons+KA and neurons +KA+astrocytes, *n* = 3 in each group). We blotted the IGF-1 and PS-198 on the same membrane as both were taken from the neurons, while the astrocytic IGF-1 was blotted to astrocytes medium. The concentration of IGF-1 secreted by neurons and associated expression of p-tau is positively correlated with KA concentration (*R*^2^ = 0.4008, *P* = 0.0271; *R*^2^ = 0.8145, *P* < 0.0001), by Pearson analysis.

While cultured neurons without astrocytes are very sensitive to acute excitotoxic insult elicited by KA (Figure 1A), when cultured with astrocytes, neurons become very resilient (Figure 1B). To confirm whether IGF-1 is involved in this effect, we first found that endogenous IGF-1 increases after KA treatment. As shown in Figure 2, only neurons secrete IGF-1 into the culture medium in a dose-dependent way according to the amount of KA (*R*^2^ = 0.4008, *P* = 0.0271); while astrocytes secrete substantial and dose-independent amounts of IGF-1 after KA (Figures 2A,B,E,F). As neuronal loss by KA has been found to be associated with hyperphosphorylated tau, and here we found that KA increased p-tau expression with a dose-dependent effect as well (*R*^2^ = 0.8145, *P* < 0.0001) and the cocultured astrocyte could reduce the p-tau regardless of KA doses (Figures 2C,D).

To explore the distinct role of neuronal and astrocytic IGF-1, we applied a PI3K-Akt chipset to KA treated neurons and co-culture system (Supplementary Figure S2).

**FIGURE 3:**
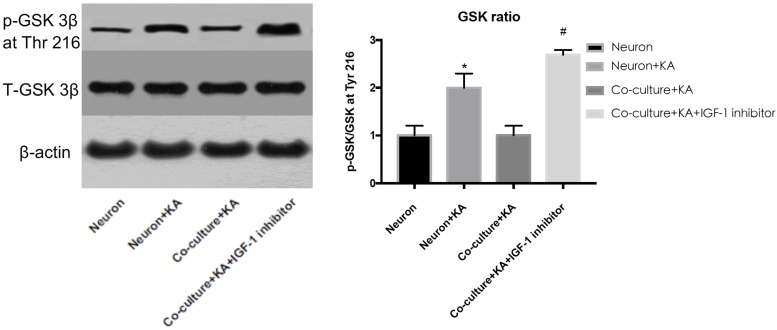
The GSK-3β activity is indicated by the ratio of p-GSK-3β (Tyr 216)/Total-GSK-3β. KA induced the increased GSK-3β activity, while co-cultured astrocyte decreased the GSK-3β activity, and this effect was blocked with astrocytic IGF-1 inhibitor (^*^P < 0.05 compared to the isolated neurons; ^#^*P* < 0.05, compared to co-culture+KA group).

Previous studies showed tau hyperphosphorylation is induced by increased activity of GSK3β (Xue et al., 2017), which is a main kinase in the body to phosphorylate tau proteins (Liu et al., 2016). Based on this, we could consider that co-cultured astrocytes decrease the expression of p-GSK3 at Tyr 216 site to decrease the GSK3β activity and further decrease hyperphosphorylated tau and protects neurons from excitotoxicity, which is confirmed by WB analysis (Figure 3 and Table 1).

**TABLE 1:** Gene changes in excitotoxic neurons with cocultured astrocytes.

**Upregulate genes**	**Log value**	**Downregulated genes**	**Log value**
Mapk14	3.200262919	Fkbp1a	−1.035214322
Mapk8	2.984302712	Csnk2a1	−1.145787057
Prkcz	2.444014809	Grb10	−1.218625033
Rps6ka1	1.925751768	Rheb	−1.285215569
Ilk	1.882264841	Pabpc1	−1.361158017
Btk	1.81595143	Rasa1	−1.367858696
Nfkbia	1.719726027	Wasl	−1.519555394
Myd88	1.445691899	Rac1	−1.554536649
Pik3cg	1.406636297	**GSK-3β**	**−1.567745615**
Tirap	1.151062562	Eif4ebp1	−2.126020794

### Impact of rAAV8-GFAP-hIGF-1 in a Brain Excitotoxicity Model

**FIGURE 4:**
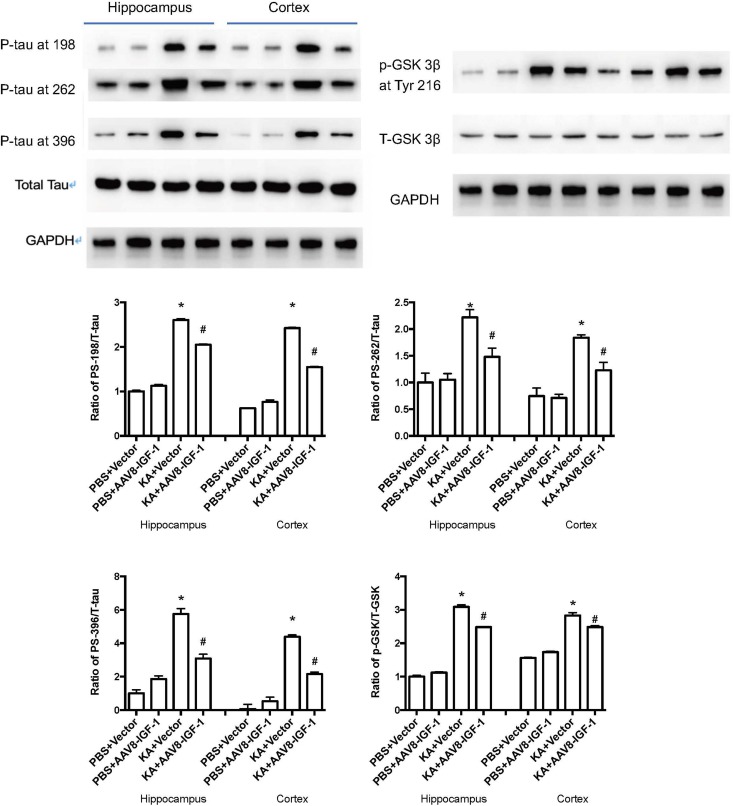
Gene transfer of astrocytic IGF-1 decreases p-tau expression at different phosphorylated sites (PS-198, 262, and 396) and reduces the ratio of GSK-3β at Tyr 216 of total GSK-3β in both cortex and hippocampus via reducing the phosphorylated GSK-3β. We calculated the ratio of both p-tau to total tau and p-GSK to total GSK, therefore, we used the same GAPDH image for the control protein loading comparison. Up panel, representative blot images. Down panel, quantification of WB results. ^*^Comparison between KA+Vector to PBS+vector (*P* < 0.05); ^#^comparison between KA+AAV8-IGF-1 to KA+Vector (*P* < 0.05). Data shown as mean SEM (*n* = 3 in each group).

Considering the role of astrocytic IGF-1 in *in vitro* studies, we accordingly explored whether increasing astrocytic IGF-1 would be essential in a brain excitotoxicity *in vivo* model. As shown in Figure 4, GFAP-AAV8 -hIGF-1 transfer reduced the p-tau in both cortex and hippocampus of KA mice at different phosphorylated sites (PS-198, PS-262, and PS396), which is associated with the decreased GSK-3β expression.

**FIGURE 5:**
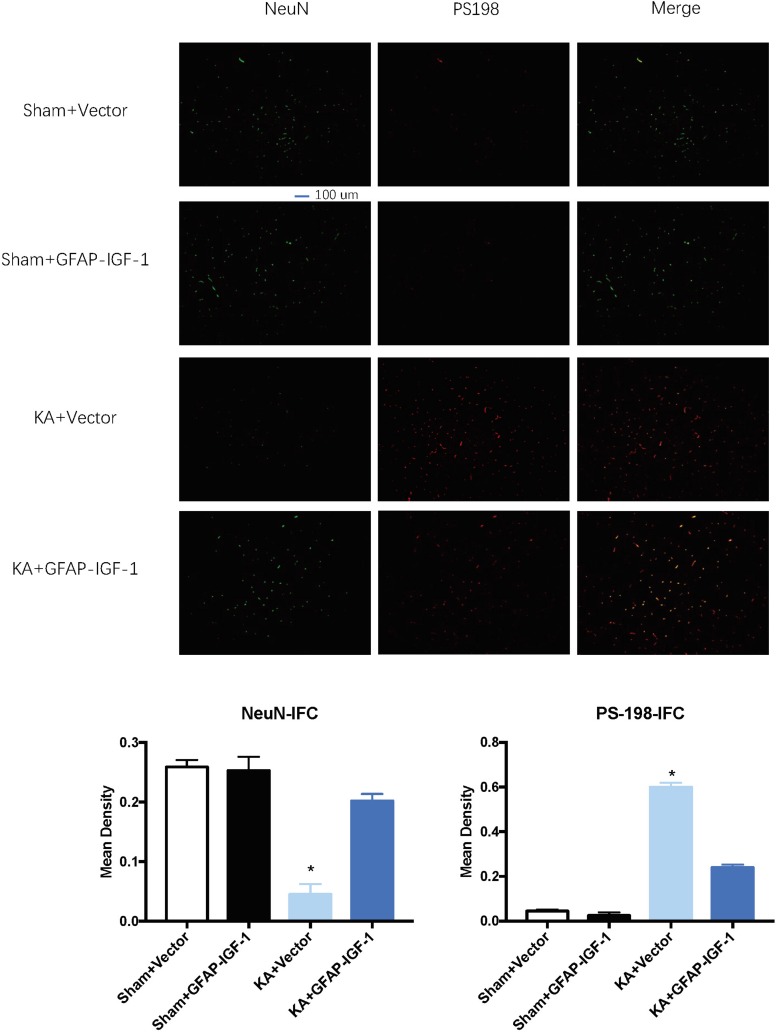
Gene transfer of astrocytic IGF-1 decreases the p-tau expression at the cortex (PS-198 fluorescence) and increases neuronal cell loss (NeuN). KA reduces the mean density of NeuN compared to sham mice, while the astrocytic IGF-1 increases the neuronal mean density. The mean fluorescence density of PS-198 also increases in KA mice, and astrocytic IGF-1 transfer decreases it. ^*^P < 0.001, opposed to other groups. *n* = 3 in each group, and data is indicated as mean ± SEM.

**FIGURE 6:**
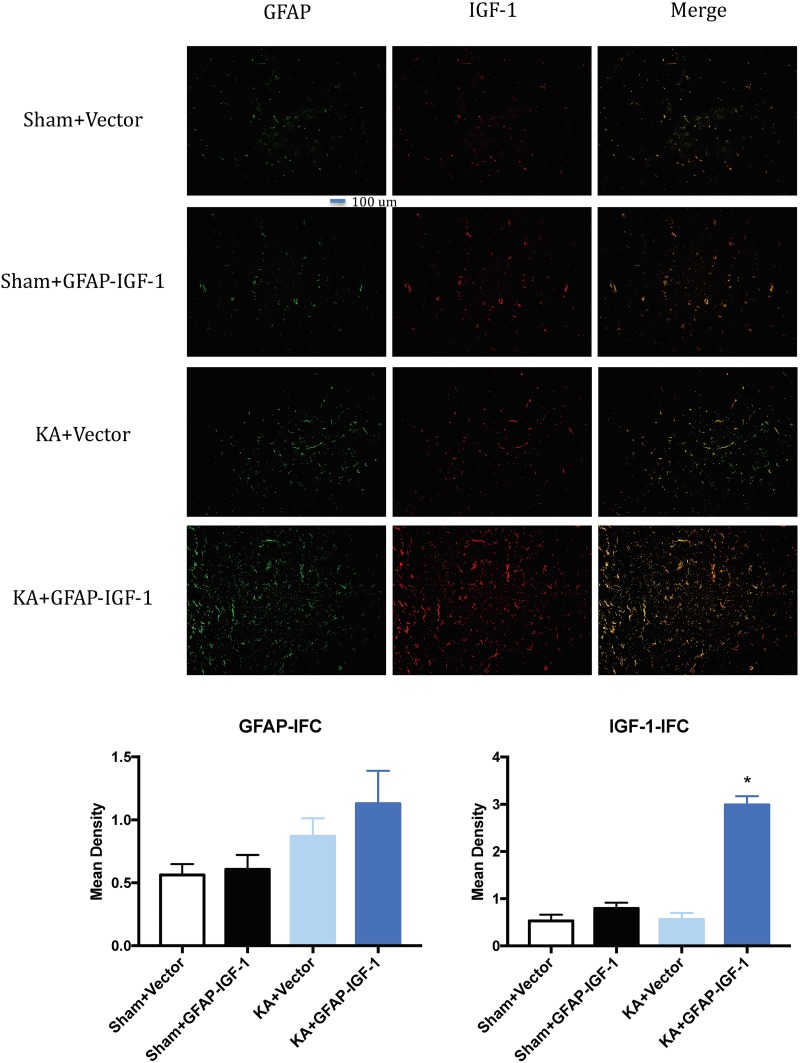
Gene transfer of astrocytic IGF-1 shows most IGF-1 co-localized with the astrocytes. There is no difference in GFAP expression among these groups. The IGF-1 is mostly increased in KA+GFAP-IGF-1 group, showing that increased IGF-1 might be due to both IGF-1 secretion from astrocytes and IGF-1 transfer from virus. ^*^P < 0.001, compared to other groups, *n* = 3 in each group, and data is indicated as mean ± SEM.

Gene transfer of IGF-1 also decreases the neuronal loss and PS-198 in cortex (indicated by fluorescence mean density, Figure 5) and most IGF-1 is colocalized with the GFAP expression (Figure 6). The increased IGF-1 might come from both viral infection and cell secretion.

### Selective Overexpression of IGF-1 in Astrocytes Improves Neuronal Dysfunction

**FIGURE 7:**
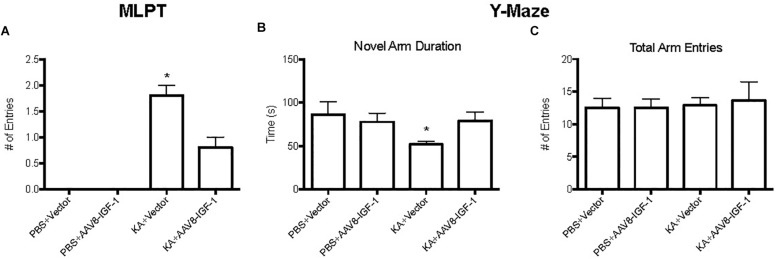
Behavior test of KA mice treated with empty vectors or hIGF-1. KA mice with empty vector showed higher MPLT score and hIGF-1 could reduce it; while KA mice with empty vector showed decreased less novel arm duration, and hIGF-1 could increase it **(A,B)**. No significant differences in total arm entries in all four groups **(C)**. ^*^P < 0.05, statistically different to other three groups, *n* = 10 in each group).

To explore the effect of replenishing astrocytic IGF-1 on neurological outcome, motor function was evaluated by modified limb placing test (MLPT) and cognitive function by a Y-Maze at 28 days post-injury. Excitotoxic brain injury lead to significant motor dysfunction as demonstrated by higher MLPT scores after KA treatment in both control and IGF-1 treated mice compared to the sham group with PBS treatment (Figure 7). Nevertheless, IGF-1 treated KA mice showed statistically lower MPLT scores compared to those mice with control vector (Figure 7A), indicating that astrocytic IGF-1 overexpression attenuated excitotoxic motor dysfunction.

Excitotoxic injury also caused an obvious cognitive deficit in KA treated mice with a 50% less preference for a novel arm (Figure 7B), while excitotoxic IGF-1 mice without a cognitive dysfunction. In addition, excitotoxic IGF-1 mice demonstrated longer duration of the novel arm compared to control mice (Figure 7B), indicating astrocytic IGF-1 replenishment prevented spatial learning dysfunction. Motor performance of all mice was equivalent, indicating the motor dysfunction was not a bias during the cognitive test (Figure 7C).

## Discussion

In the present study, we analyzed the neuroprotective role of endogenous astrocytic IGF-1 after KA -induced neurodegeneration. We found KA resulted in hyperphosphorylation of tau and neuronal death in both *in vivo* and *in vitro* studies. When neurons were co-cultured with astrocytes, there was a lesser degree of neuronal loss and decreased tau phosphorylation and this rescuing effect was blocked with astrocytic IGF-1R antagonist from *in vitro* studies.

Our findings show that neurons in the presence of astrocytes are less sensitive to KA excitotoxicity, as evidenced by decreased MTT in co-cultured neurons. This finding extended the recent finding of cell-specific neuroprotection of IGF-1 signals. These observations highlight the importance of cell-specific IGF-1 against excitotoxic challenge. Therefore, a better understanding of the cell-specific role of IGF-1 in the brain requires considering its effects on other cells in brains which need further studies to investigate the relationship between astrocytes and microglia and oligodendrocytes.

A lower sensitivity of cocultured neurons with astrocytes to KA provided by IGF-1 allows these cells to survive against excitotoxicity challenge. While in response to excitotoxic injury, the production of IGF-1 by cultured astrocytes and neurons is increased, consistent with the report that after brain ischemia IGF-1 levels are actually higher due to increased synthesis and accumulation in microglia, vessels and astrocytes (Beilharz et al., 1998). Therefore, *in vivo*, astrocytes and neurons will receive IGF-1 input from various local sources, suggesting that the response of increased IGF-1 after brain insults reflects an endogenous neuroprotective mechanism against brain insults (Zheng and Tong, 2017). This conclusion is apparently consistent with previous evidence that genetic ablation of IGF-1 in mice, increases p-tau levels in the brain (Cheng et al., 2005). However, mice with reduced IGF-1 activity (hemizygous for the IGF-1 receptor) have lower levels of Aβ and diminished neuroinflammation in the brain (Kappeler et al., 2008; Nadjar et al., 2009). And these mice exhibited improved spatial memory and reduced anxiety (De Magalhaes Filho et al., 2017). Conceivably, the effects of modulating IGF-1 signaling prior to brain insult (as when using genetic models) may not be the same as after an insult. For example IGF-1 protects nerve cells and/or the brain against diverse types of excitotoxicity-related insults (Puche et al., 2008; Grinberg et al., 2012). However, exogenous IGF-1 applied in TBI could induce hyperactivity in rodents (Song et al., 2016). In this regard, we emphasize the importance not only of cell type but also of context dependency of IGF-1 neuroprotection in relation to excitotoxicity.

A role for excitotoxicity in many neurodegenerative diseases is gaining increasing acceptance (Zheng et al., 2014). Aberrant production of p-tau in the central nervous system is linked to neurodegenerative diseases such as Alzheimer Disease dementia, Parkinson’s disease, traumatic brain injury, epilepsy or stroke, all of them associated to aging and neurodegeneration (Zheng et al., 2014). However, as already commented, the role of excitotoxicity in brain aging is still unclear (Shultz et al., 2015). An attempt to explain these apparently opposing observations is that increased hyperphosphorylated tau levels may activate neurodegenerative pathways (Liu et al., 2016). The present findings confirm this proposal. Thus, doses of KA up to 100 M do not elicit astrocyte death probably because IGF-1 helps maintain their anti-degenerative capacity and at the same time their neuroprotective action. In this regard, our results show that astrocytes in response to KA activate IGF-1-PI3K-Akt signaling including upregulation of IGF-1 coupled to downregulation of phosphorylated GSK-3β at Tyr216. GSK-3β has several phosphorylation sites and the phosphorylated tyrosine kinase 216 can increase its activity to hyperphosphorylated tau proteins, which is a hallmark in AD, and the p-tau plays an important role in different brain diseases in which excitotoxicity is implicated. The fact that co-localization shows p-tau mostly expressed in neurons reinforces results that have been proved by a series of studies previously (Padmanabhan et al., 2006; Quintanilla et al., 2009, 2012). Tau proteins can be phosphorylated at different sites with a profile of phosphorylation, which indicated the extent of p-tau. In our study, we found KA induced p-tau in 198,262 and 396 sites and the increased IGF-1 in astrocytes could reduce these p-tau at separate sites.

Adeno associated virus has been used in clinical session to treat patients. rhIGF-1 has a neuroprotective function, however, it can cause the hyperexcitability and post-traumatic epilepsy. The injection of the AAV: GFAP-IGF-1-GFP could infect the GFAP positive neurons and increase the expression of IGF-1 in astrocytes (shown by Figure 6). In this study, we applied gene transfer of hIGF-1 with a GFAP promoter to transfect astrocytes *in vivo* and found hIGF-1 could prevent the motor and cognitive dysfunction in KA mice. And this might be translated to clinical studies for patients with neurodegenerative-like behavior.

**FIGURE 8:**
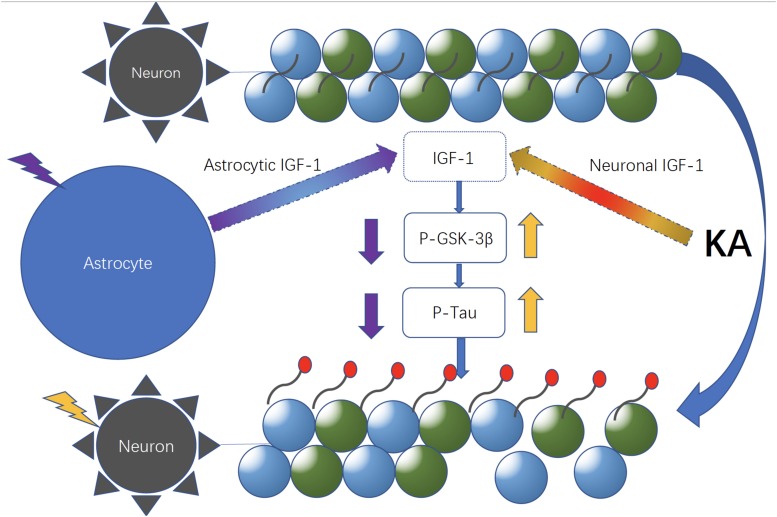
Neuronal and astrocytic IGF-1 might have different roles in excitotoxicity. Neuronal and astrocytic IGF-1 seem to have different effects in excitotoxicity. When neurons alone are treated with KA, they are prone to be hyperphosphorylated with increased GSK-3β activity; in contrast, while neurons are co-cultured with astrocytes, they are resilient to KA and become less phosphorylated due to secretion of astrocytic IGF-1.

According to the PCR array and WB result, co-cultured astrocyte secreted IGF-1, which can further decrease the GSK-3β activity indicated by the decreased ratio of p-GSK-3β (Tyr 216)/Total-GSK-3β. And this effect was blocked with astrocytic IGF-1 inhibitor (AG1024 added in the astrocyte medium). IGF is found to stimulate glycogen synthase mainly mediated via the signaling cascade PI(3)K/Akt/GSK-3β that leads to the inhibited GSK3 kinase activity. However, total GSK-3β expression did not change following KA or co-culture. In this case, we thought inconsistent expression of decreased GSK-3β RNA and normal total GSK-3β protein expression might be due to the GSK-3β alternative splicing which was affected by IGF-1, according to a previous report (Wang et al., 2012). Nevertheless, the alternative splicing of GSK-3β needs further study to confirm it. This might explain the fact that the cell-specific IGF-1 has different roles in neuroprotection after excitotoxic injury. Our findings show that specific increased IGF-1 in astrocytes can prevent neurological degeneration after TBI. However, exogenous IGF-1 treatment following TBI would result in neuronal hyperactivity, and the exogenous IGF-1 might change the original context such as astrocytes function and characteristics in brains and this needs further exploration with electrophysiological studies. Here, we found that neuronal IGF-1 increased after KA treatment with a dose-response effect, while astrocytes can also release IGF-1 after KA but with a consistent concentration, which further decreased p-tau expression via decreasing the GSK-3β activity. The intracellular mechanisms mediating neuroprotection of the endogenous cell-specific IGF-1 against excitotoxicity involve GSK-3β, a kinase phosphorylates tau proteins. These findings indicate astrocytic but not neuronal IGF-1 has a neuroprotective effect in excitotoxicity. Neuronal and astrocytic IGF-1 seem to have different effects in excitotoxicity. When neurons alone are treated with KA, they are prone to be hyperphosphorylated with increased GSK-3β activity; by contrast, while neurons are co-cultured with astrocytes, they are resilient to KA and become less phosphorylated due to secretion of astrocytic IGF-1 (Figure 8). And it would be useful to investigate the structural difference of IGF-1 secreted from neurons or astrocytes, due to the fact that IGF-1 has different isoforms in neurons and astrocytes, respectively, and these isoforms may have different biological effects. In summary, cell specific IGF-1 in brain responses to excitotoxicity challenge underscores the need to design therapeutic strategies that consider all aspects of biological organization, leading, for example, to cell-specific targeting of anti-aging drugs.

## Conclusion

Taken together, our data demonstrate that astrocyte-derived IGF-1 has a beneficial effect on function and pathology following an excitotoxic injury. Importantly, we show that the IGF-1 reduces not only neuronal apoptosis but also expression of phosphorylated tau via regulating GSK-3β. Thus, the action of the IGF-1 is multifold and impacts a number of cell survival and regulatory pathways both *in vitro* and *in vivo*. As cell-specific IGF-1 shows tremendous promise as therapeutics, understanding the downstream and upstream pathways is critical for understanding their mechanism of action as well as determining how to apply the cell-specific IGF-1 with the most potential to benefit.

## Data Availability

The datasets supporting the conclusions of this article are available from the corresponding author.

## Ethics Statement

The animal study was reviewed and approved by the local ethics committee in Shanghai Pudong New area People’s Hospital, Shanghai University of Medicine and Health Sciences (20170223-001).

## Author Contributions

PZ and WC conceived the study, designed the experiments, and performed the cell cultures. WT and JZ ran the molecular tests. PZ and BH wrote the manuscript. All authors read and approved the final manuscript.

## Conflict of Interest Statement

The authors declare that the research was conducted in the absence of any commercial or financial relationships that could be construed as a potential conflict of interest.

## Supplementary Material

FIGURE S1 Neuronal culture images show the neural outgrowth in primary cortical neurons.

FIGURE S2 PCR-array results for PI3k-Akt pathway in co-culture system and neuron only after KA.

## Abbreviations

GSK-3β: glycogen synthase kinase
IGF-1: insulin-like growth factor-1
KA: kainic acid
MLPT: modified limb preference test
MTT: thiazolyl blue tetrazolium bromide.
