# Supplementary figures and images for: Astrocytic Insulin-Like Growth Factor-1 Protects Neurons Against Excitotoxicity
Source: Front Cell Neurosci. 2019 Jul 9;13:298. doi: 10.3389/fncel.2019.00298 (PMC6629877; doi:10.3389/fncel.2019.00298)

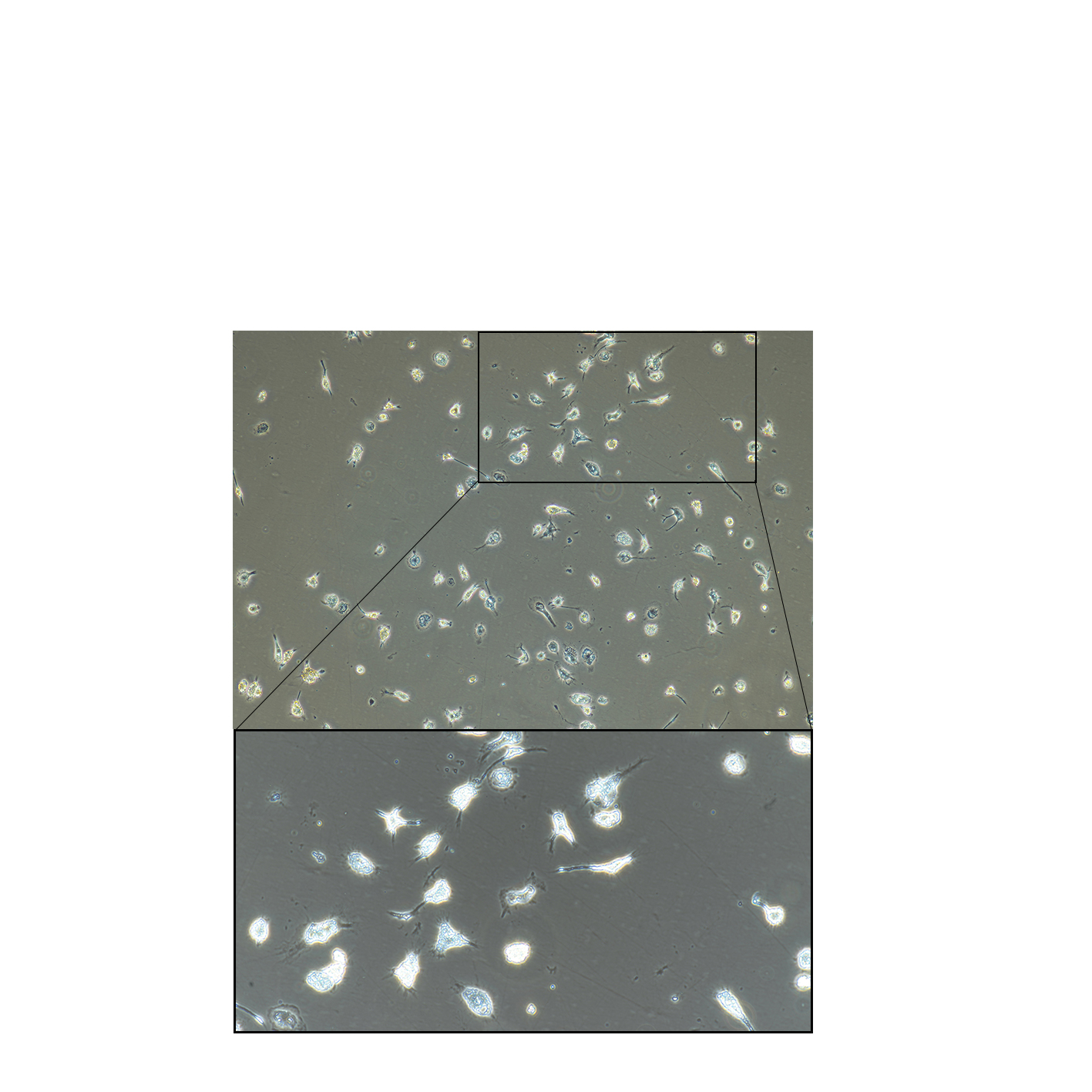

Supplement: FIGURE S1 — Neuronal culture images show the neural outgrowth in primary cortical neurons. [file Image_1.jpeg]

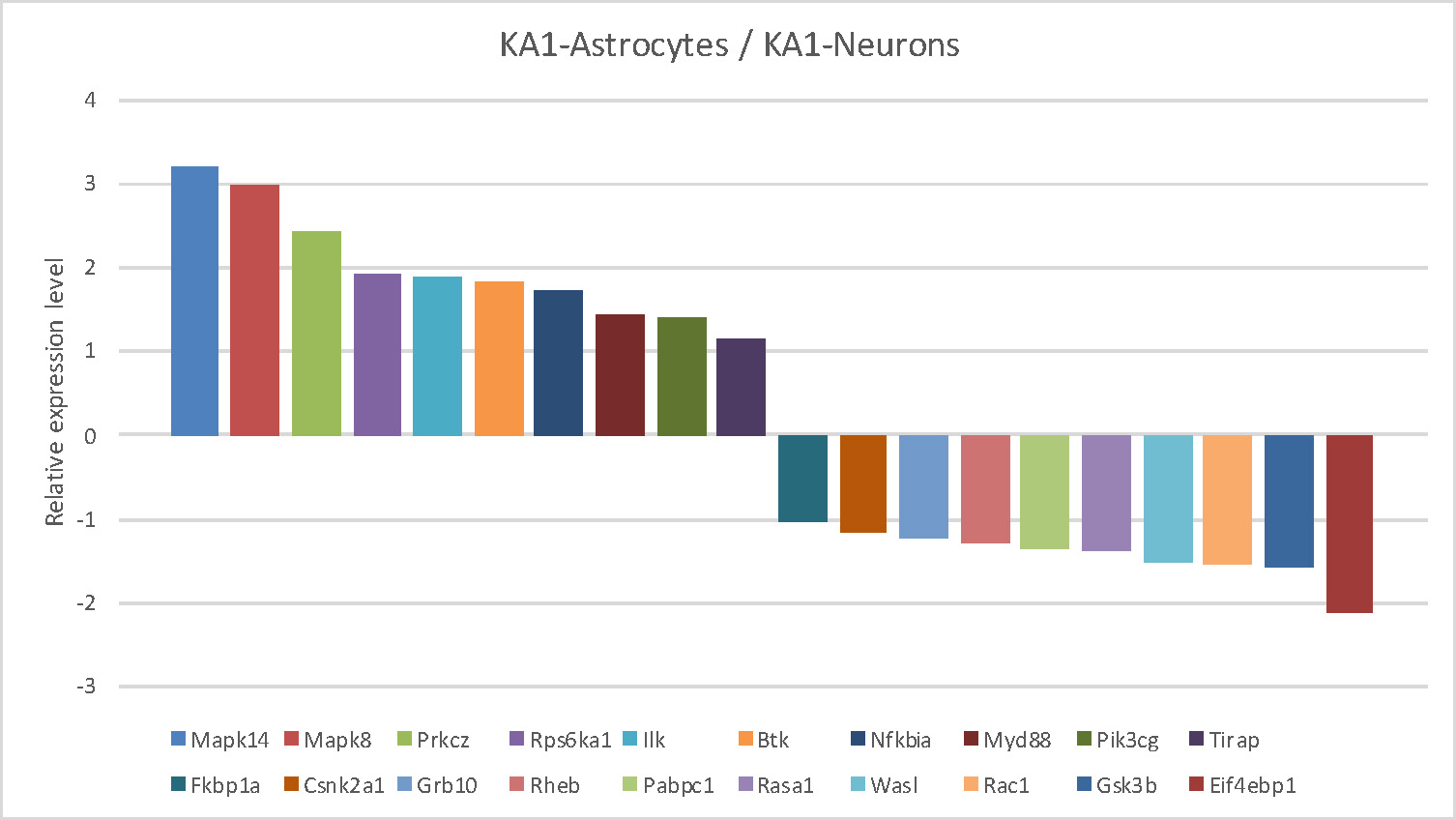

Supplement: FIGURE S2 — PCR-array results for PI3k-Akt pathway in co-culture system and neuron only after KA. [file Image_2.jpeg]
